# A Paraganglioma in the Right Supraclavicular Fossa: Mistaken for a Thyroid Mass

**DOI:** 10.7759/cureus.13773

**Published:** 2021-03-08

**Authors:** Elias Nikolopoulos, Katherine Ploumidou, Antigoni Sourla, Christos Kittas

**Affiliations:** 1 Department of Otolaryngology, Head and Neck Surgery, Omilos Iatrikou Athinon, Athens, GRC; 2 Department of Otolaryngology, Children’s Hospital Aglaia Kiriakou, Athens, GRC; 3 Department of Pathology and Laboratory Medicine, Bioiatriki Laboratories, Athens, GRC; 4 Department of Pathology and Laboratory Medicine, Ethnikon Kai Kapodistriakon Panepistimio Athinon, Athens, GRC

**Keywords:** paraganglioma, ectopic thyroid mass, supraclavicular fossa

## Abstract

Paragangliomas are rare lesions of the endocrine system that can be treated either by embolization preoperatively followed by surgical excision or by surgical excision or radiotherapy. In this report, we present an extremely rare location of a nonfunctional paraganglioma in the head and neck region, located in the right supraclavicular fossa, which was misdiagnosed as a thyroid tumor, in a 72-year-old female patient. Imaging revealed a 2.5 × 4.5 × 2 cm well-defined vascularized mass. Fine-needle aspiration (FNA) on the tumor was not diagnostic initially; however, a third attempt revealed thyroid cells suggesting the existence of an ectopic thyroid tumor. FNA was additionally performed on the right thyroid lobe, revealing atypical follicular colloid cells of the Bethesda 3 category. Therefore, the excision of the ectopic thyroid tumor along with right lobectomy was planned. No embolization was initiated preoperatively in this case. Histopathology revealed that the supraclavicular mass was a paraganglioma. Paragangliomas supplied by the subclavian, innominate, and common carotid artery are rarely reported, and to our knowledge, this is the third case to be reported worldwide.

## Introduction

Paragangliomas are highly vascularized tumors that emanate from the paraganglia of neural crest cells and appear commonly in the head and neck region; they are classified as symptomatic or asymptomatic based on their ability of endogenous hormone secretion [1,2]. They usually occur in the carotid body [3-7], jugulotympanic area [8-12], and vagal paraganglia [4,13]; they are also found in other regions in the head and neck such as the thyroid [2,3,6,10], nasal cavity [11], endolarynx [12], and supraclavicular fossa [1].

The prevalence of paragangliomas in the head and neck region is 0.03%. They usually become obvious in the fifth or sixth decade of life and are more prevalent in females than males. Carotid body tumors, glomus jugulare tumors, and vagal paragangliomas account for 98% of this malignancy. Paragangliomas in other regions of the neck are extremely rare, especially those found in the supraclavicular fossa [1].

Most paragangliomas include neurosecretory granules and appear as a painless asymptomatic mass, although 1-3% of them may be symptomatic and may clinically manifest symptoms resembling those of a pheochromocytoma due to catecholamine secretion [4]. Positron emission tomography (PET)/CT has been highlighted as useful in the diagnosis of paragangliomas; however, immunohistochemistry remains the gold standard of the primary diagnostic method [2]. Fine-needle aspiration (FNA) biopsy is commonly non-diagnostic for these tumors and can be misleading and may lead to the condition being misinterpreted as medullary thyroid cancer [6]. Treatment modalities include surgery, with or without presurgical embolization, and radiotherapy [4].

About 75% of these tumors are sporadic and 25% are hereditary. Mutations of the gene, which converts into a coded form the succinate dehydrogenase (SDH) enzymes SDHD, SDHA, SDHB, and SDHC, are known to cause familial head and neck paragangliomas. Mutations in the SDHB are the reason for familial adrenal pheochromocytoma and extra-adrenal paraganglioma in the thorax and abdomen region. The current biological understanding of paragangliomas holds that they are tumors of deceptive behavior. Therefore, the classification of paragangliomas as benign tumors is debatable [4].

## Case presentation

**Figure 1:**
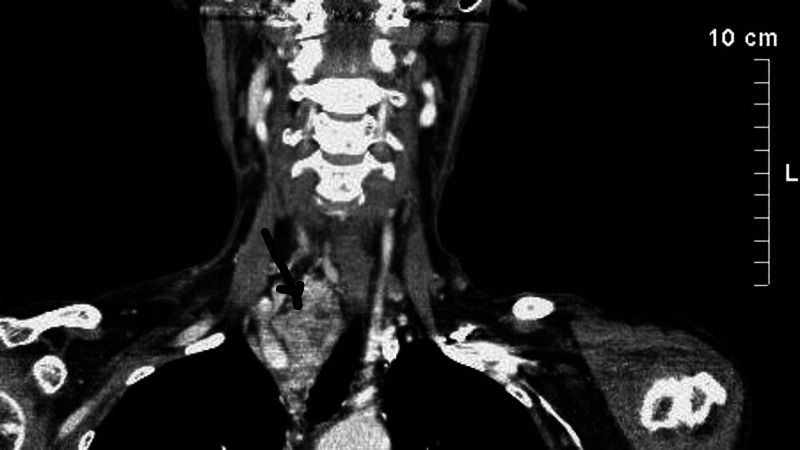
Three-dimensional CT angiography revealing blood supply of the mass The arrow indicates the position of the mass in the right supraclavicular fossa CT: computed tomography

**Figure 2:**
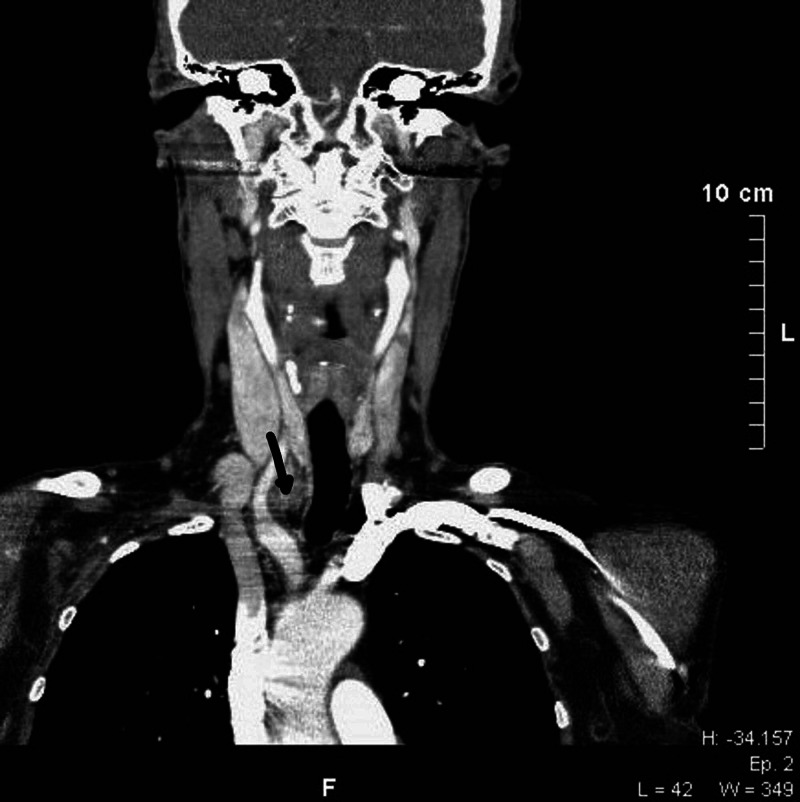
Three-dimensional CT of the tumor The arrow indicates the position in close proximity with the innominate artery CT: computed tomography

A 72-year-old female with an unremarkable medical history presented to the Department of Otolaryngology-Head and Neck Surgery at Athens Medical Center (AMC), with a history of an eight-month-long right swelling in the region of the supraclavicular fossa. No other clinical symptoms were mentioned. Clinical examination revealed a firm painless mass. Examination with rigid endoscopes was unremarkable. The patient underwent neck ultrasonography, which displayed a multinodular thyroid of 0.1-0.3 cm nodules and a well-defined mass of 4.5 x 2.5 x 2 cm situated in the lower pole of the right thyroid lobe. CT showed a vascular mass in the right supraclavicular region and in close proximity to the lower pole of the right lobe of the thyroid. Furthermore, three-dimensional CT angiography demonstrated a vascularized mass mainly supplied by the subclavian artery, the common carotid, and the innominate (Figures 1, 2).

The results of two attempts of FNA on the mass were inconclusive while the third attempt suggested the presence of an ectopic thyroid tumor. FNA on the thyroid was also conducted, which revealed atypical follicular colloid cells of the Bethesda 3 category. Vanyllylmandelic acid was not examined. Thyroid hormone levels were within the normal range. Taking these findings together, we presumed it was an ectopic thyroid tumor in close proximity to the lower pole of the right lobe. The surgical plan consisted of a right hemithyroidectomy together with full excision of the presumed ectopic thyroid tumor. Preoperative embolization was not carried out. Surgery was successfully executed, even though aggressive intraoperative bleeding occurred during the dissection of the mass. Due to the proximity of the tumor to major vessels, a vascular surgeon was present during the operation. Postoperative recovery was uneventful with no complications. After pathological examination and verification of histological results, which provided a surprise finding, epinephrine and norepinephrine were examined and were found within normal ranges. A PET-CT was performed two months after the surgery and revealed the absence of residual tumor. Genetic testing revealed no relationship with any SDH mutations.

## Discussion

**Figure 3:**
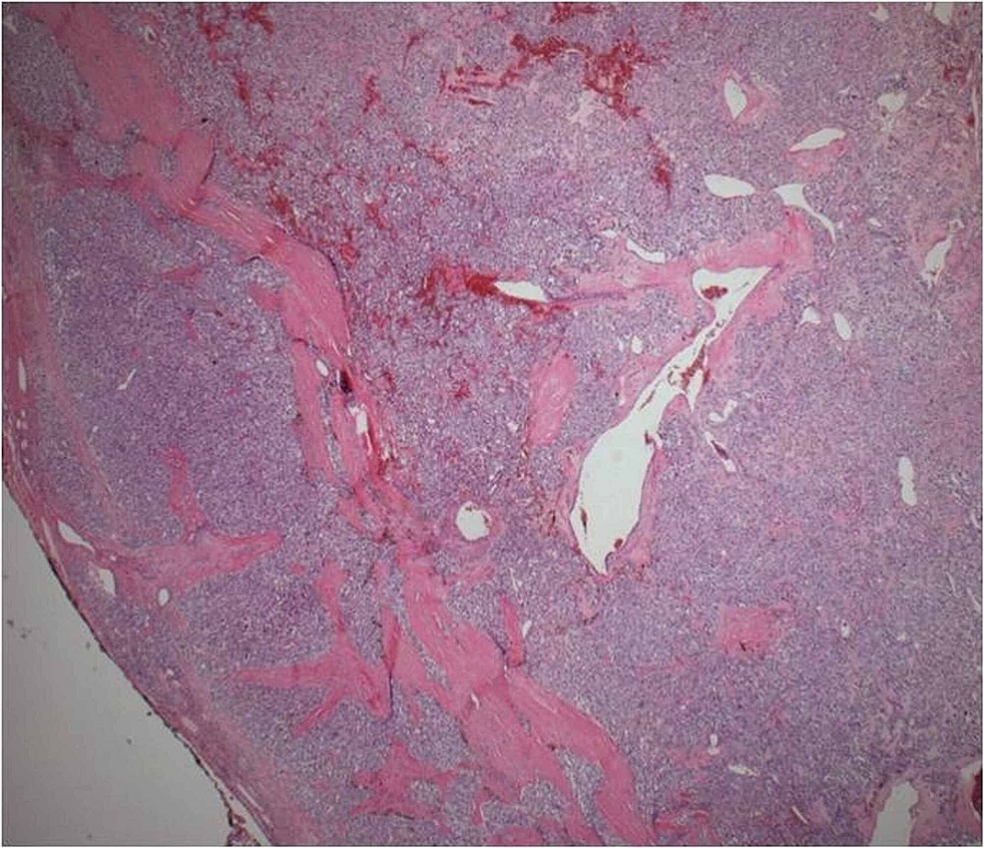
Nests of cells (zellballen) with vascular stroma with thin fibrous capsule

On macroscopic examination for both frozen biopsy and routine histopathological evaluation, an oval, relatively circumscribed tissue specimen of 4.5 x 2.5 x 2 cm in total dimensions with a thin fibrous capsule was found and examined. On cut sections, a neoplastic mass of 2.4 cm of maximum diameter was observed. The cut surface was brownish with whitish areas and of soft and focally loose-cystic consistency. The right thyroid lobe weighed 5.7 grams and was 4.2 x 2 x 1.9 in total dimensions with a reddish-brownish cut surface, of elastic consistency, and with many colloid nodules. On microscopic examination, histologically, the neoplasm consisted of nests of cells (zellballen) with eosinophilic and/or clear granular cytoplasm in a fine vascular stroma (Figure 3).

**Figure 4:**
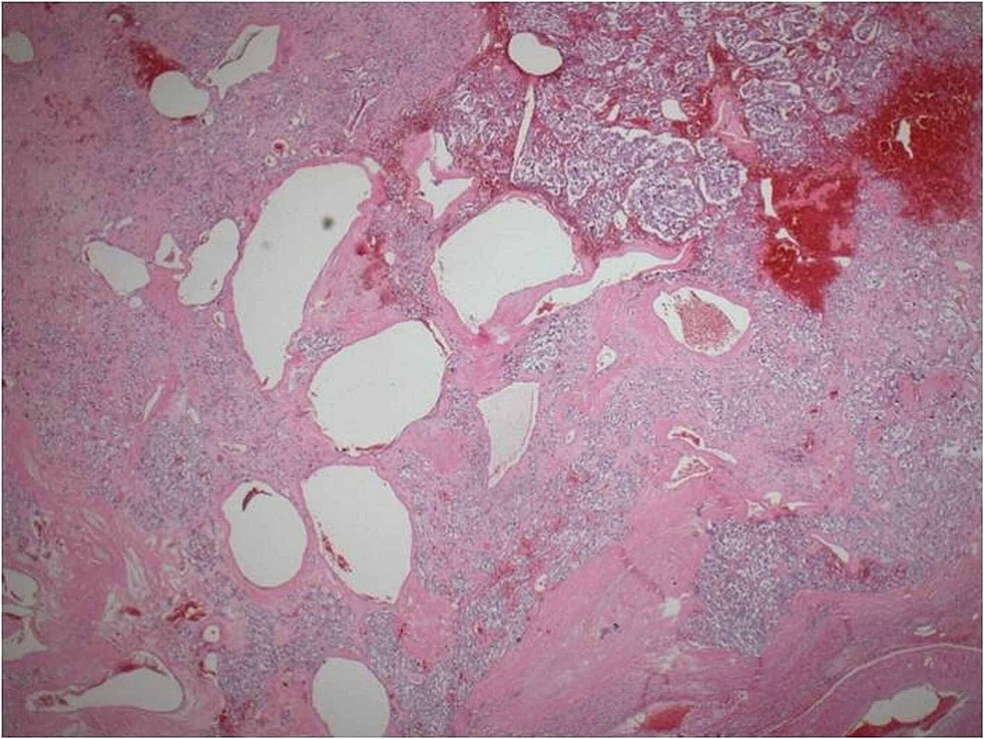
Nests of cells areas of hemorrhage and cystic spaces

Atypical cells with large hyperchromatic nuclei without the presence of mitoses or necrosis were observed. There were areas of hemorrhage and cystic degeneration of the neoplasm as well as vascular congestion and hyalinization of the stroma (Figure 4).

**Figure 5:**
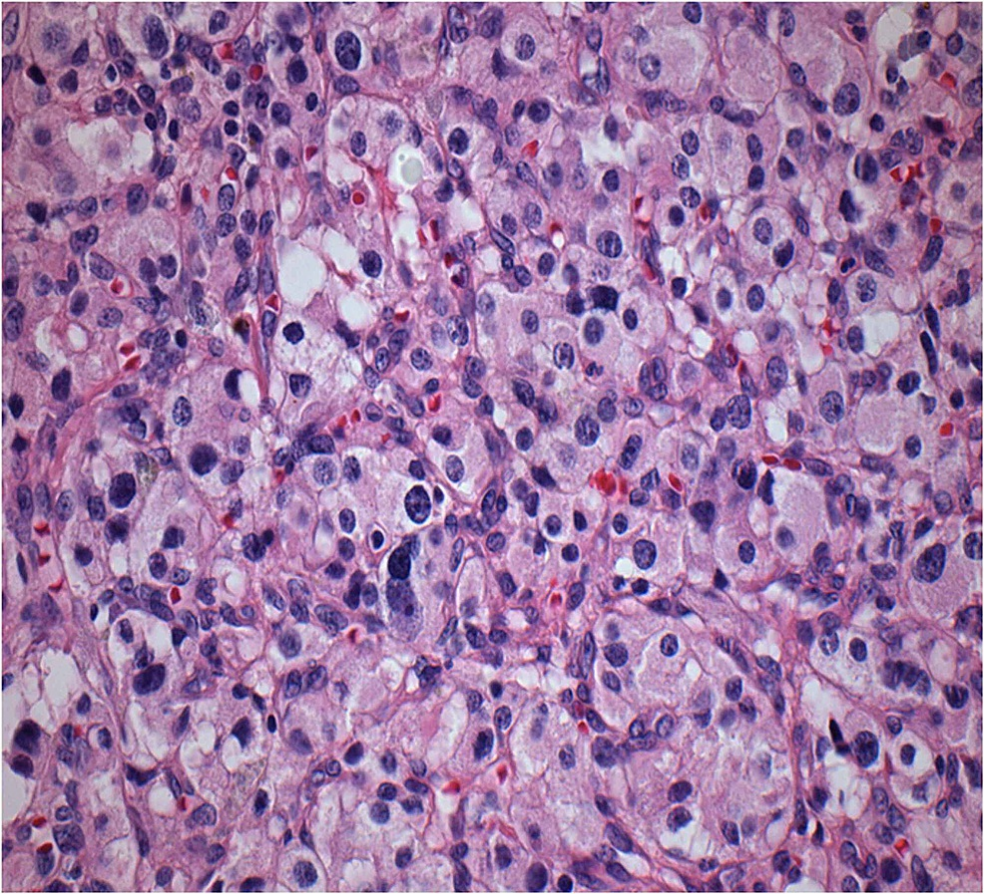
Cells with eosinophilic/clear cytoplasm and cells with nuclear atypia

**Figure 6:**
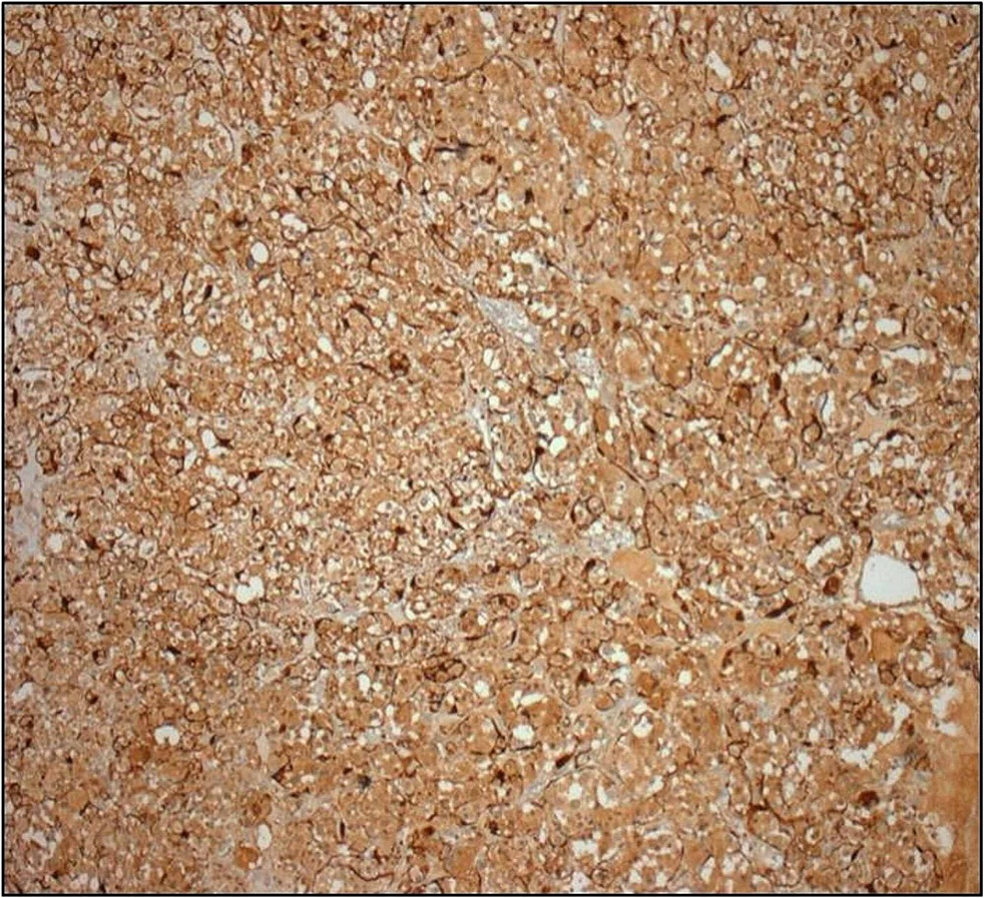
Immunohistochemistry - chromogranin

**Figure 7:**
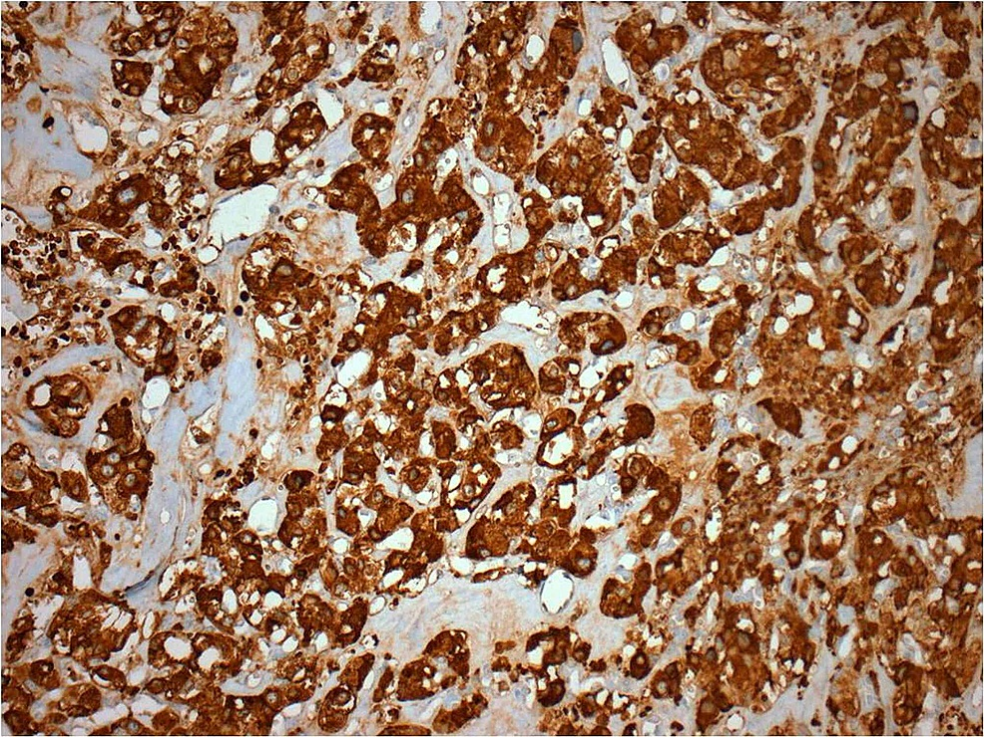
Immunohistochemistry - S100

**Figure 8:**
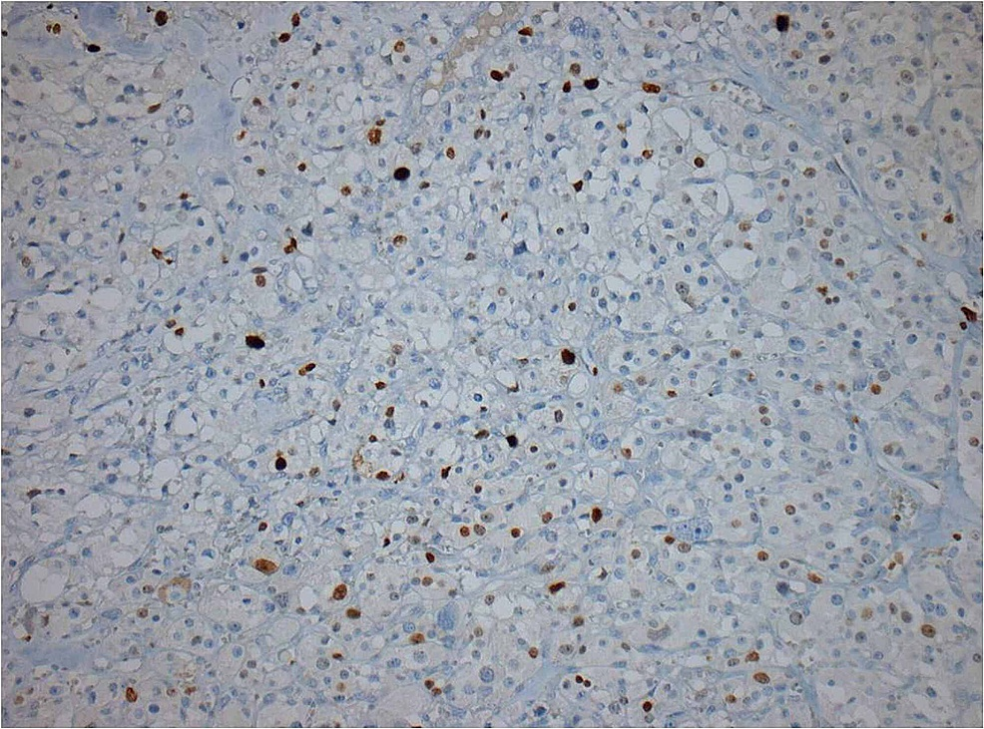
Ki67 with a proliferation index of 10%

The neoplasm was surrounded by a thin fibrovascular capsule being in continuity with fibrovascular tissue containing medium-sized veins and nerves. Immunohistochemistry revealed the positivity of neoplastic cells (Figure 5) in synaptophysin, chromogranin (Figure 6), and S100 (Figure 7), whereas the markers pankeratin and CD10 were negative. Proliferation marker Ki-67 (Figure 8) was positive in 10% of neoplastic cells.

Iron histochemical stain revealed foci of hemosiderin depositions in areas of hemorrhagic infiltration. The thyroid parenchyma demonstrated features of nodular hyperplasia with many colloid nodules of 0.1-0.3 in maximum diameter and a mild chronic monocytic inflammation. The pathological department concluded that it was a tumor of the right cervical area with histomorphological and immunohistochemical features compatible with a cervical paraganglioma of 2.4 cm in maximum diameter. The surgical margins, as well as the fibrovascular capsule of the neoplasm, were free of neoplastic infiltration.

Paragangliomas emanate from the parasympathetic ganglia, composed of either chromaffin or non-chromaffin cells. These tumors are categorized as symptomatic or asymptomatic based on whether endogenous catecholamine hormone excretion is elaborated. CT, CT angiography, MRI, and PET/CT have been found useful for the diagnosis of the disease [1,2]. Specifically, Ga-labelled somatostatin agonist PET is favored regardless of the patient's genetic background [13]. Fine-needle biopsy plays an elusive role in the diagnosis of paragangliomas, and cytology is non-diagnostic most of the time [2,6].

Broadly, it is well known that malignant paragangliomas are often associated with lymph nodes or distant metastasis [2]; nevertheless, the current biological understanding of paragangliomas holds that they are tumors with unpredictable, elusive behavior and should not be categorized as benign. While all SDH genes are associated with a risk for metastasis, SDHB-mutated tumors constitute the highest risk for metastasis, with their association with metastasis ranging from 30 to 50%. Thus, the overall survival is quite poor, ranging from 11 to 36%. Surgery, although challenging due to the tumor's close proximity to vital vascular structures and cranial nerves [4], still remains the most common treatment method for paragangliomas. Complete resection of the lesion is a critical component of remission [2] and the only method that can ensure complete removal of the disease. Patients with bilateral lesions and positive family history should be referred for genetic analysis [5]. The entity of SDHB mutations should alert surgeons to proceed with early and extensive surgery, a fact of paramount importance for patient's safety and overall survival [4]. Unfortunately, tumors related to SDH mutations display more aggressive behavior [5]. If a paraganglioma tumor had not been diagnosed preoperatively, patients should postoperatively undergo hormonal evaluation for functional disease, imaging evaluation for multi-centric and metastatic disease, as well as genetic counseling [3].

Both primary thyroid paragangliomas and cervical paragangliomas imitating thyroid nodules have been described in the literature. These tumors present a diagnostic dilemma. Neck paragangliomas are usually nonfunctional, and if located near the thyroid gland, they usually present as a slightly enlarging, solid hypervascular nodule of the thyroid gland. Specifically, paragangliomas can be misjudged for medullary thyroid cancer when localized close to or within the thyroid gland, due to similar microscopic features on cytology [6]. Currently, there is no definitive histological method to distinguish benign from malignant paragangliomas. The appearance of atypia in tumor cells does not necessarily indicate malignancy; however, obvious necrosis and the presence of mitotic features in the center of the tumor crest, vascular invasion, and capsular status are important signs for prognosticating malignant features [2]. Nevertheless, histological evaluation is unable to provide a definitive diagnosis of malignancy [5]. For this reason, lifelong follow-up is crucial for this patient population [4].

Preoperative embolization is therefore particularly helpful in large and hypervascular tumors (Shamblin Ⅱ and Ⅲ), and in some practices, embolization is chosen to be carried out, a day before surgery, using non-reabsorbable agents such as polyvinyl alcohol (PVA) particles [5].

Conventionally, apart from radionuclide therapy administration of alkylating agents or tyrosine kinase inhibitors (13), the nonsurgical treatment of paragangliomas entails recent methods of radiotherapy, which involve single-fraction gamma knife radiosurgery and CyberKnife (Accuray Incorporated, Sunnyvale, CA) radiosurgery [8,9,13].

## Conclusions

We presented a case of an asymptomatic paraganglioma in the supraclavicular fossa supplied mostly by the subclavian artery, which was mistaken for a thyroid tumor. The latter was located beneath the nearest segment of the right carotid artery in the neck and was unrelated to the vagus nerve. At the same time, a right hemithyroidectomy was integrated into the surgical procedure. A neck mass at the supraclavicular fossa area is seldom identified as a paraganglioma. This fact should be taken into account in the differential diagnosis of neck masses in the specific region of the neck.
